# Gut metagenomes of type 2 diabetic patients have characteristic single-nucleotide polymorphism distribution in *Bacteroides coprocola*

**DOI:** 10.1186/s40168-017-0232-3

**Published:** 2017-02-01

**Authors:** Yaowen Chen, Zongcheng Li, Shuofeng Hu, Jian Zhang, Jiaqi Wu, Ningsheng Shao, Xiaochen Bo, Ming Ni, Xiaomin Ying

**Affiliations:** 1 Beijing Institute of Basic Medical Sciences, Beijing, 100850 People’s Republic of China; 2 Beijing Institute of Radiation Medicine, Beijing, 100850 People’s Republic of China

**Keywords:** Type 2 diabetes, Metagenome, Bacteria, Phylogenetic analysis, SNP enrichment

## Abstract

**Background:**

Gut microbes play a critical role in human health and disease, and researchers have begun to characterize their genomes, the so-called gut metagenome. Thus far, metagenomics studies have focused on genus- or species-level composition and microbial gene sets, while strain-level composition and single-nucleotide polymorphism (SNP) have been overlooked. The gut metagenomes of type 2 diabetes (T2D) patients have been found to be enriched with butyrate-producing bacteria and sulfate reduction functions. However, it is not known whether the gut metagenomes of T2D patients have characteristic strain patterns or SNP distributions.

**Findings:**

We downloaded public gut metagenome datasets from 170 T2D patients and 174 healthy controls and performed a systematic comparative analysis of their metagenome SNPs. We found that *Bacteroides coprocola*, whose relative abundance did not differ between the groups, had a characteristic distribution of SNPs in the T2D patient group. We identified 65 genes, all in *B. coprocola*, that had remarkably different enrichment of SNPs. The first and sixth ranked genes encode glycosyl hydrolases (GenBank accession *EDU99824.1* and *EDV02301.1*). Interestingly, alpha-glucosidase, which is also a glycosyl hydrolase located in the intestine, is an important drug target of T2D. These results suggest that different strains of *B. coprocola* may have different roles in human gut and a specific set of *B. coprocola* strains are correlated with T2D.

**Electronic supplementary material:**

The online version of this article (doi:10.1186/s40168-017-0232-3) contains supplementary material, which is available to authorized users.

## Background

Human gut microbiota are critical to human health and have been related to various disease conditions, such as obesity [1], diabetes [2–4], cirrhosis of the liver [5], inflammatory bowel disease [6], atopic dermatitis [7], and pulmonary inflammation [8]. Next-generation sequencing (NGS) and bioinformatics technologies provide access to the genetic information of the entire microbiome and thus enable systematic investigation of its composition and functional genetics.

A number of metagenomics studies have compared the relative abundance of bacterial species or pathway enrichment between patients and healthy controls (HCs). However, little work has been done to elucidate strain-level variations or single-nucleotide polymorphisms (SNPs) in the metagenome. Because even slight nucleotide variations can alter the pathogenic behavior and antibiotic resistance of bacteria [9–11], analyses of genomic variations (i.e., SNPs, insertions, and deletions) and structural variation of the metagenome are important for understanding microbiome biology.

Regarding strain-level variation in the microbial metagenome, Schloissnig and colleagues proposed a workflow for analyzing metagenomics datasets at the strain level and described the genomic variation landscape of human gut microbial genomes [12]. Subsequently, Greenblum et al. detected extensive variations in strain-level copy-numbers in the human gut microbiome [13] and Zhu et al. reported considerable differences (based on gene deletions) in the gene content of strains within the same species in the human gut [14]. Tropism and persistence of different oral Neisseria strains have also been studied using metagenomics sequencing [15]. Although genomic variation of microbiomes is well documented by these studies, association of strain-level metagenomics findings with human diseases has been limited.

Type 2 diabetes (T2D) is a complex metabolic disorder afflicting hundreds of millions of people worldwide [16]. Because T2D is related to diet and digestion, the role of gut microbiota in T2D initiation and progression is of great interest. Previous large-scale T2D-related metagenomics research has shown that the proportion of phylum *Firmicutes* and class *Clostridia* cells in the microbiome is significantly reduced in T2D [2] and that the gut microbiota of T2D patients tends to have fewer butyrate-producing bacteria, such as *Roseburia intestinalis* and *Faecalibacterium prausnitzii* [3].

To the best of our knowledge, no prior study has resolved the association between T2D and the gut microbiome at a strain or SNP level. Here, we utilized a public NGS dataset resource and performed a comparative study examining the SNPs of gut metagenomes in T2D patients relative to HCs.

## Methods

### T2D and control dataset

Raw NGS datasets of DNA obtained from fecal samples of 170 T2D patients and 174 HCs in China [3] were downloaded from the NCBI Sequence Read Archive (total data, 1.2 terabases; average sample size, 3.5 gigabases; accession numbers SRA045646 and SRA050230). To reduce SNP false positives, we trimmed abnormal bases and filtered low-quality reads as described in detail previously [12]. For each base (A, T, G and C), a mean number of base calls (*f*) across all sites and a standard deviation (SD) were calculated. Starting from the first site at 5′ end, the site was trimmed if the base call number for a base was beyond *f* ± 2 × SD. The trimming at 5′ end was terminated until encountering a site with all base call numbers within the range. Next, Trimmomatic [17] was used to remove adapters and trim low-quality bases (<Q20) at the 3′ end (parameters: -phred33 ILLUMINACLIP:adapters.fa:1:0:7 TRAILING:20 SLIDINGWINDOW:5:10 MINLEN:45 AVGQUAL:20). A total of 1.04 terabases of data remained after quality control procedures were completed. Clinical information and other characteristics of the 344 individuals included in the analysis were obtained from [3] and [18].

### Bacterial reference genome determination

We first determined a set of bacterial reference genomes for samples included in this study. Metaphlan2, which is based on approximately 17,000 reference genomes [19], was employed to profile the bacterial species in each sample. Genomes of species that were identified by metaphlan2 in at least four samples were included in the reference set for the alignment analyses. The genome sequences of all of these species were downloaded from the NCBI assembly database and are listed in Additional file 1: Table S1.

### Selecting species and genes with sufficient supporting reads

Clean reads were aligned to the whole reference set with Burrows-Wheeler Aligner-maximal exact match (BWA-MEM) [20] in default settings, and only unique alignments were outputted. Species with sufficient supporting reads were selected with a cutoff of ≥40% of the reference genome being covered by a ≥10× depth in at least 20 samples in both the T2D and the control groups. For genes, the cutoff was ≥80% gene sites with ≥10× depth in at least 10 samples in each group. Only the species and genes that met these criteria were subjected to subsequent SNP analyses.

### SNP and intra-sample variation calling and filtering

Two tools, BCFtools [21] and VarScan2 [22], were applied to identify SNPs of the metagenome. The alignment of duplicates by BWA-MEM was first marked and filtered in Picard [23]. Then, SAMtools [24] was used to generate “mpileup” files from the SAM-formatted alignment files. The mpileup files were employed as input files for both BCFtools and VarScan2. The parameters used for BCFtools and VarScan2 were “-vmO z –V indels” and “pileup2snp min-coverage 10, *p* value 0.05, min-avg-qual 15,” respectively. SNPs detected by both the tools were selected. SNPs were further filtered with the requirements of a ≥0.5 mutated allele (relative to that in the reference genome) frequency, ≥4× supporting reads for the variant, and strand bias of sequencing bases less than tenfold in both BCFtools and VarScan2. Namely, when genome sites with heterozygosity were found in a sample, the major allele was used for the SNP analysis.

To address the heterozygosity or intra-sample variations, mutated allele frequencies (MuAFs) of variant sites were obtained for analysis under polyclonal scenario. The MuAFs of sites were defined as the mean values of outputs by BCFtools and VarScan2, which were highly correlated (*R* ^2^ = 0.997). To examine whether intra-sample variations affect the result, SNPs with a >0.8 MuAF were selected for a parallel SNP analysis.

### Annotation of genes and SNPs

Gene ontology (GO) annotations of each genome were downloaded from Uniprot [25]. SNPs were annotated in SnpEff [26] with the -eff parameter. The genome annotation files used by SnpEff were obtained from GenBank.

### Phylogenetic tree construction based on whole-genome level SNPs of *B. coprocola*

Based on the reference genome of *B. coprocola* (reference strain DSM 17136, GenBank accession *GCA_000154845.1*), genome regions with >20% samples not having valid coverage (≥10× depth) were discarded. If ≤20% of the samples had invalid coverage in a region, the bases in that region in those samples were labeled as “N.” Then, the nucleotides at SNP sites from the samples were extracted to generate a pileup file. Phylogenetic trees were constructed based on the whole-genome level aligned SNPs by using randomized axelerated maximum likelihood (RAxML) v8.2.9 (100 bootstrap replicates), with GTR model of nucleotide substitution, γ-distributed rates among sites, and Felsenstein correction for ascertainment bias [27, 28]. The parameters for RAxML were “-#100 –m ASC_GTRGAMMA –f a.” Rooting was undertaken by using RAxML with “-f I” option. Trees were drawn with the R package ggtree [29].

### Phylogenetic tree construction of genes

The nucleotide sequences of genes were obtained by aligning reads to corresponding full-length genes of the reference genome. For a given gene, gene regions were discarded if with >20% samples not having valid coverage (≥10× depth) and the coverages of full-length genes are listed in Additional file 1: Table S4. If ≤20% of the samples had invalid coverage in a region, the bases in that region in those samples were labeled as “N.” Then, the full-length genes, except the discarded regions, were aligned. The phylogenetic trees of genes were constructed by using RAxML v8.2.9 with settings as these for whole-genome level, but without ascertainment correction. The two clusters are separated at the root of the phylogenetic tree.

### Clustering of samples by mutated allele frequencies of sites

For a given samples, the sites of bacterial genome and gene with a >0.2 MuAFs were selected. For all samples, we obtained a matrix with the column denoting site positions and the rows denoting samples. If there was no variation or MuAFs <0.2, the MuAFs were set to 0. Then, hierarchical clustering and affinity propagation (AP) clustering [30] were applied to the matrix. The hclust function of stat package in R v3.3.0 was used for hierarchical clustering with parameters “method = complete,” and the output tree was drawn by ggtree. For AP clustering, APcluster in R v3.3.0 was employed with parameters “*K* = 2.” The clusters generated by AP clustering were visualized by using Cytoscape v3.5.0 [31]. For each node, the top five edges connecting the nodes with the largest similarity are shown.

### Statistical analysis

Relative abundance of species and genome/gene densities were compared between the T2D group and control group with the Mann-Whitney test. Fisher’s exact test was performed to test for bias of SNP sites and sample enrichment inferred from the phylogenetic tree and clusters by AP clustering. Hypergeometric test was applied for one-tailed test for enrichment of biased SNP sites at gene level. The *q* value with Storey and Tibshirani’s method (R package qvalue v1.43.0) was applied for multiple testing correction [21] to identify species and genes with significantly biased SNP distribution.

## Results

Based on gut metagenomics data from 344 individuals (170 T2D patients and 174 healthy controls), we identified a total of 356 bacterial species (Additional file 1: Table S1; Additional file 2: Figure S3) and their relative abundances. Consistent with previous reports, we found that, relative to the HC group, the T2D group had lower proportions of phylum *Firmicutes*, class *Clostridia,* and butyrate-producing bacteria (Additional file 2: Figure S1) [2, 3]. Relative abundance did not differ between the groups for 270 of the 356 species (75.8%) analyzed (Mann-Whitney test, *p* > 0.05; Additional file 1: Table S2).

We selected 20 bacterial species with sufficiently supporting NGS reads in a sufficient number of samples (see “Methods” section, Additional file 1: Table S3) for analysis of SNP distribution. Based on the reference genomes of these 20 species, a total of 5.94 million SNPs were identified, of which 99.65% were bi-allelic and 0.35% were tri-allelic. The distributions within these 20 species of the normalized bi-allelic SNP densities calculated for genome regions with valid coverage (>10×) in the T2D and HC groups are reported in Additional file 2: Figure S2. The SNP density distribution differed significantly between the T2D and HC groups (Mann-Whitney test, *p* = 0.0083, *q* = 0.0258) for only one of the 20 species, namely, *Bacteroides coprocola* (reference strain DSM 17136, Genbank accession *GCA_000154845.1*). However, the mean relative abundance of *B. coprocola* for the T2D group (9.10 ± 7.09%) was similar to that for the control group (8.91 ± 7.16%; Mann-Whitney test, *p* = 0.9646) in the samples with sufficient reads. Moreover, *B. coprocola* was prevalent and identified in 31.98% (110/344) of all the samples, ranked the top 24th among the 356 species.

**Fig. 1:**
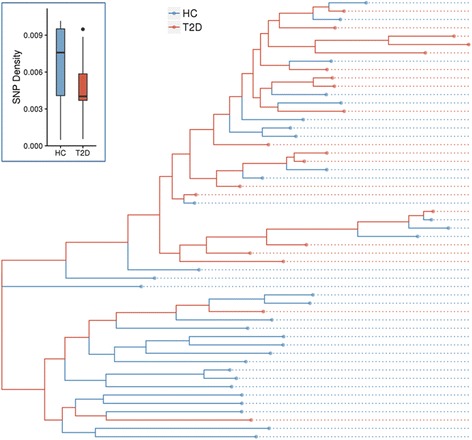
Phylogenetic tree based on *B. coprocola* SNPs. *Red* and *blue branches* indicate T2D samples and HC (i.e., normal) samples, respectively. The boxplot graph of SNP density for *B. coprocola* in each sample group is shown in the *inset graph*

A phylogenetic tree of the samples that was constructed based on the *B. coprocola* SNPs revealed a biased distribution of T2D patients versus HCs (Fig. 1). The intra-tree distance (quantified based on average pairwise patristic distance) among T2D individuals’ genomes (0.0079) was smaller than that of controls (0.0109) and that determined for the total sample pool (0.0103). We further examined variation distribution of *B. coprocola* under polyclonal scenario. We found that 94.00% of variations in *B. coprocola* had a >0.8 MuAF (Additional file 2: Figure S4). We also built a phylogenetic tree based on SNPs with >0.8 MuAFs (Additional file 2: Figure S5) and clustered samples by MuAFs of variations (Additional file 2: Figure S6). The results are consistent and imply that T2D patients may share a specific set of *B. coprocola* strains.

Examination of the SNP distributions of the protein-coding genes of the selected 20 gut bacteria revealed that 51,579 genes in the 20 microbe species had valid coverage with sufficient prevalence (see “Methods” section). Among them, we identified 1300 genes (2.52%) with significantly differentiated SNP densities between T2D and control samples (Mann-Whitney test, *q* < 0.05; Additional file 1: Table S4). All but one of these genes were found in *B. coprocola*, whose reference genome contains 4291 protein-coding genes. The one gene (*EFQ08025.1*) not in *B. coprocola* was from *Faecalibacterium cf. prausnitzii*. With the reference strain of *B. coprocola* DSM 17136, we observed that generally there are more SNPs in the HC group compared to those in the T2D group at the gene level (1268/1300, Additional file 1: Table S4).

**Fig. 2:**
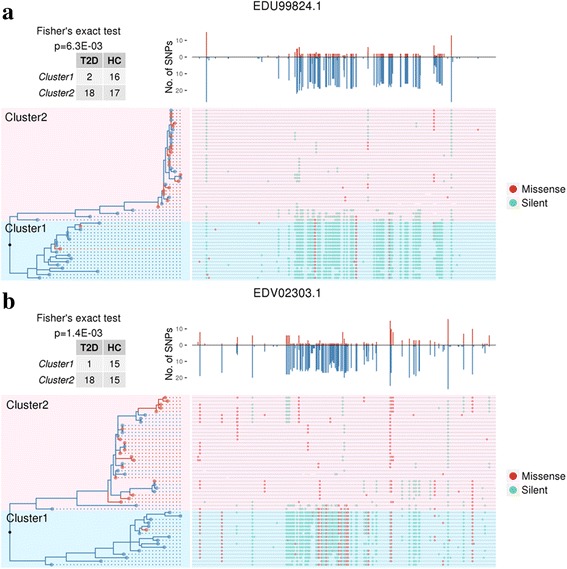
Phylogenetic trees and SNP distributions of the genes *EDU99824.1* (**a**) and *EDV02303.1* (**b**). Samples in clusters 1 and 2 are indicated by *light blue* and *pink shading*, respectively. The *lines* aligned to tree leaves represent corresponding gene sequences with sufficiently covered reads, with missense (*red dot*) and silent (*green dot*) SNPs indicated. The *bar graphs* above the gene sequences show the number of SNPs found at each site (aligned to each bar) in the T2D group (*red bars* above the axis) and the HC group (*blue bars* below the axis). Fisher’s exact test results for 2 × 2 contingency tables are shown in the *upper left* of each panel

To select the genes with the most differentiated SNP distributions, we examined the group bias of each SNP site (Fisher’s exact test, *p* < 0.05) and identified 65 *B. coprocola* genes with significant enrichment of biased SNPs against the 1300 genes as a background (hypergeometric test, *q* < 0.05). Phylogenetic trees constructed based on the nucleotide sequences of the 65 genes are shown together with their associated gene SNP distributions; the top two genes with the most differentiated SNP distribution are shown in Fig. 2 and the rest of the genes in Additional file 3. Interestingly, we observed clear assemblage of the trees for 49 of these 65 genes into two distinct clusters. The T2D samples enriched in one cluster and the other dominantly consisted of HC samples (Fisher’s exact test, *p* < 0.05). The most biased gene encodes a glycosyl hydrolase (GenBank accession *EDU99824.1*) with a biased SNP ratio of 0.59, and the second encodes a response regulator receiver domain protein (GenBank accession *EDV02303.1*) with a ratio of 0.48. Both of these genes had a biased distribution of T2D versus HC samples in the two tree clusters (Fisher’s exact test, *p* < 0.05). For example, 88.89% (16 of 18) and 93.75% (15 of 16) of cluster 1 for the genes *EDU99824.1* and *EDV02303.1* were from HCs, respectively. Meanwhile, T2D samples were enriched preferentially in the second cluster (18 of 20 for *EDU99824.1* and 18 of 19 for *EDV02303.1*). We further found that 9.08% of the SNPs in *EDU99824.1* and 26.46% of the SNPs in *EDV02303.1* were non-synonymous, and non-synonymous SNPs were differentially distributed between the two clusters (Fisher’s exact test, *p* = 0.02431, *p* = 1.97E−09, respectively). Similar to genome-level analysis under polyclonal scenario, the samples were clustered by MuAFs of each gene. The results also indicated the enrichment of T2D samples in one of the clusters (Additional file 1: Table S5; Additional file 2: Figure S7–S8 for top 2 genes; Additional file 4 for the rest 63 genes). The annotations of the 65 genes with significant enrichment of biased SNP sites between the T2D and control groups are shown in Additional file 1: Table S4. Interestingly, the products of 25 genes (25/65, 38.46%) are annotated to be localized to the cell membrane.

## Conclusion

The present analysis of a Chinese metagenomics dataset revealed that the gut microbiota of T2D patients and HC individuals had different SNP distributions. The gut microbe species *B. coprocola*, which had a similar relative abundance between the T2D and HC groups, exhibited biased SNP distribution at both the genome and the gene level. Our phylogenetic analysis yielded 49 *B. coprocola* genes that had characteristic SNP distribution in T2D patients. Two of these genes (*EDU99824.1* ranked first and *EDV02301.1* ranked sixth) encode glycosyl hydrolases. Glycosyl hydrolases of bacteria play vital roles in degradation of cellulose and starch and hence generate sugars. Interestingly, alpha-glucosidase, which is also a glycosyl hydrolase located in the brush border of the small intestine, is an important drug target of T2D. Therefore, it is possible that *EDU99824.1* and *EDV02301.1* in T2D-related strain and control strain may have different glycosyl hydrolase properties, which is worthwhile to be investigated in the future.

*B. coprocola* was previously reported to have high SNP density in the gut microbiota [12]. But the correlation between its SNPs and diseases had not been investigated. Our results indicate that a specific set of *B. coprocola* strains may be associated with T2D and further suggest that strain-level bacterial colonization of the gut and the potential restorative influence of probiotic supplements should be investigated in T2D therapeutic research.

As is shown, intra-sample variations affect very slightly on our results. However, assuming one strain type per sample may not be general, and intra-sample variations should not be overlooked in strain-level analysis of metagenomics.

## Additional files

Additional file 1: Table S1. The taxonomic lineages of the 356 bacterial species and accessions of their reference genome assemblies. Table S2. Species with significantly different abundance profiles between the T2D and HC groups. Table S3. Detailed descriptive information for 20 bacterial species with sufficiently supporting NGS reads in a sufficient number of samples. Table S4. Summary of the 1300 genes found to have significantly different SNP densities between the T2D and HC groups. The top 65 genes with the most differentiated SNP distributions between the groups are highlighted in light green. Table S5. Intra-tree distances and enrichment analysis based on phylogenetic trees, hierarchical clustering trees, and affinity propagation clustering for the selected 65 genes. Table S6. SNP densities, intra-tree distances, and numbers of the most biased genes under different mutated allele frequency (MuAF) thresholds (>0.2, >0.5, and >0.8). (XLS 766 kb)
Additional file 2: Figure S1. Validation of relative abundance distribution in the T2D and HC groups. Figure S2. Comparisons of SNP densities in 20 gut bacterial species between the T2D and normal groups. Figure S3. Brief flowchart for bioinformatics analysis of metagenomics NGS data at strain level. Figure S4. Distributions of MuAFs at variant sites of *B. coprocola* in T2D (A), HC (B) and all (C) samples with sufficient NGS reads. Figure S5. Phylogenetic tree of *B. coprocola* strains based on variant sites with >0.8 MuAFs. Figure S6. Results of AP clustering and hierarchical clustering based on MuAFs of variant sites in *B. coprocola*. Figure S7. Distributions of MuAFs at variant sites in *EDU99824.1* (A) and *EDUV02303.1* (B). Figure S8. Results of AP clustering and hierarchical clustering based on MuAFs of variant sites. (PDF 761 kb)
Additional file 3: Phylogenetic trees of the 63 genes, except the top two, of the 65 genes with the most differentiated SNP distribution. Each tree is in a separate JPG file named by the GenBank accession of the corresponding gene. (RAR 13817 kb)
Additional file 4: Results of AP clustering, MuAF distributions and hierarchical clustering for 63 genes in the most differentiated SNP distribution except the top two genes. For a given gene, the clusters of AP clustering, MuAF distribution, and hierarchical tree are presented in separated files named by its GenBank accession, within the folders of APclusterTrees, MuAFhistogram, and HlustTrees, respectively. For gene *EDV02481.1*, samples were failed to be clustered into two clusters, so it has no AP clustering result. (RAR 14532 kb)

## Abbreviations

GO: Gene Ontology
HC: Healthy control
NCBI: National Center for Biotechnology Information
NGS: Next-generation sequencing
SNP: Single-nucleotide polymorphism
T2D: Type 2 diabetes
