# Supplementary material for: Gut metagenomes of type 2 diabetic patients have characteristic single-nucleotide polymorphism distribution in Bacteroides coprocola
Source: Microbiome. 2017 Feb 1;5:15. doi: 10.1186/s40168-017-0232-3 (PMC5286682; doi:10.1186/s40168-017-0232-3)
Supplement: Additional file 2: — Figure S1. Validation of relative abundance distribution in the T2D and HC groups. Figure S2. Comparisons of SNP densities in 20 gut bacterial species between the T2D and normal groups. Figure S3. Brief flowchart for bioinformatics analysis of metagenomics NGS data at strain level. Figure S4. Distributions of MuAFs at variant sites of B. coprocola in T2D (A), HC (B) and all (C) samples with sufficient NGS reads. Figure S5. Phylogenetic tree of B. coprocola strains based on variant sites with >0.8 MuAFs. Figure S6. Results of AP clustering and hierarchical clustering based on MuAFs of variant sites in B. coprocola. Figure S7. Distributions of MuAFs at variant sites in EDU99824.1 (A) and EDUV02303.1 (B). Figure S8. Results of AP clustering and hierarchical clustering based on MuAFs of variant sites. (PDF 761 kb) [file 40168_2017_232_MOESM2_ESM.pdf]

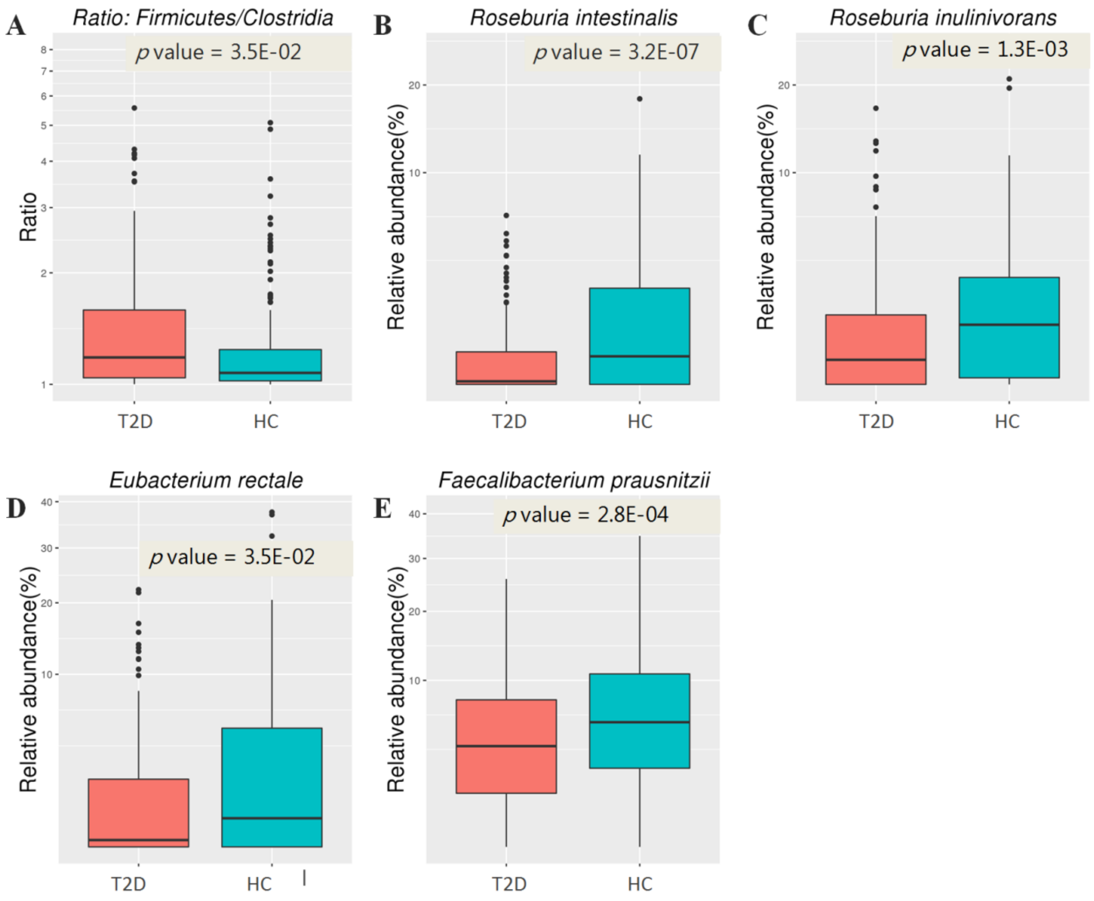


**Figure S1. Validation of relative abundance distribution in T2D and HC groups.** Boxplots of bacterial species abundance distribution are shown, with Mann-Whitney tests *p* values. **A:** Ratio of phylum *Firmicutes* abundance to that of class *Clostridia*. **B-E:** Several butyrate-producing bacteria species (labeled at the top of each panel) were less abundant in the T2D group than in the normal (*i.e.* healthy control) group. The *y* axis is logarithmic scaled.

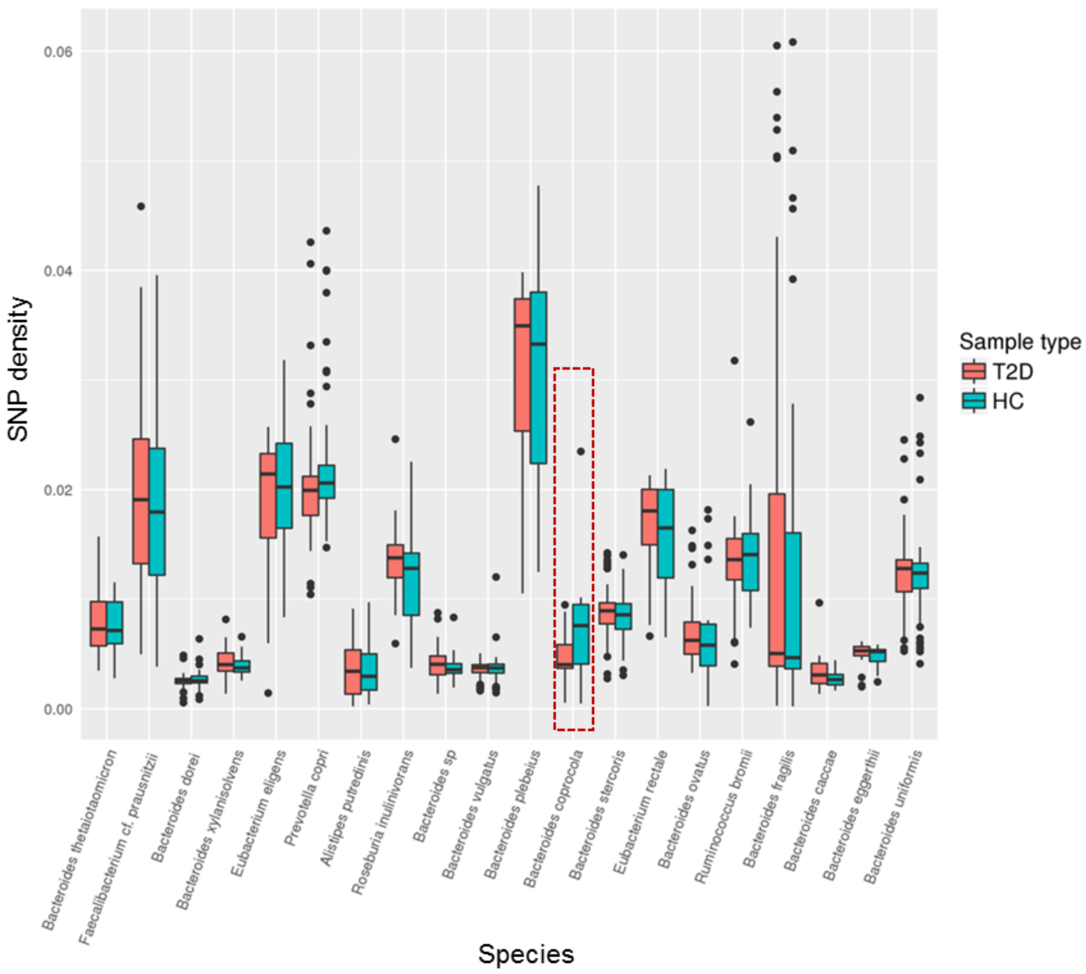


**Figure S2. Comparisons of SNP densities in 20 gut bacterial species between the T2D and normal groups.** The SNP density distribution for *B. coprocola* (red dotted box) differed significantly between the T2D and HC groups ($q$ value = 0.026).

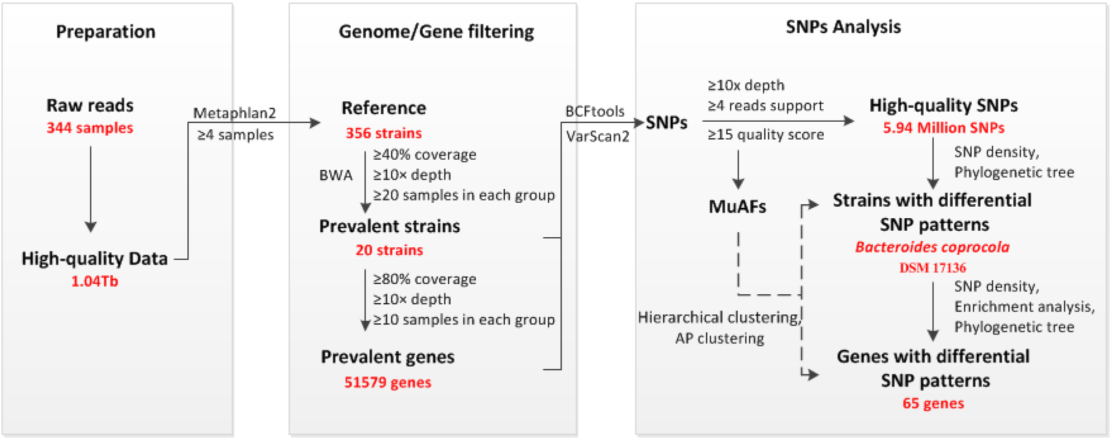


**Figure S3. Brief flowchart for bioinformatics analysis of metagenomics NGS data at strain level.**

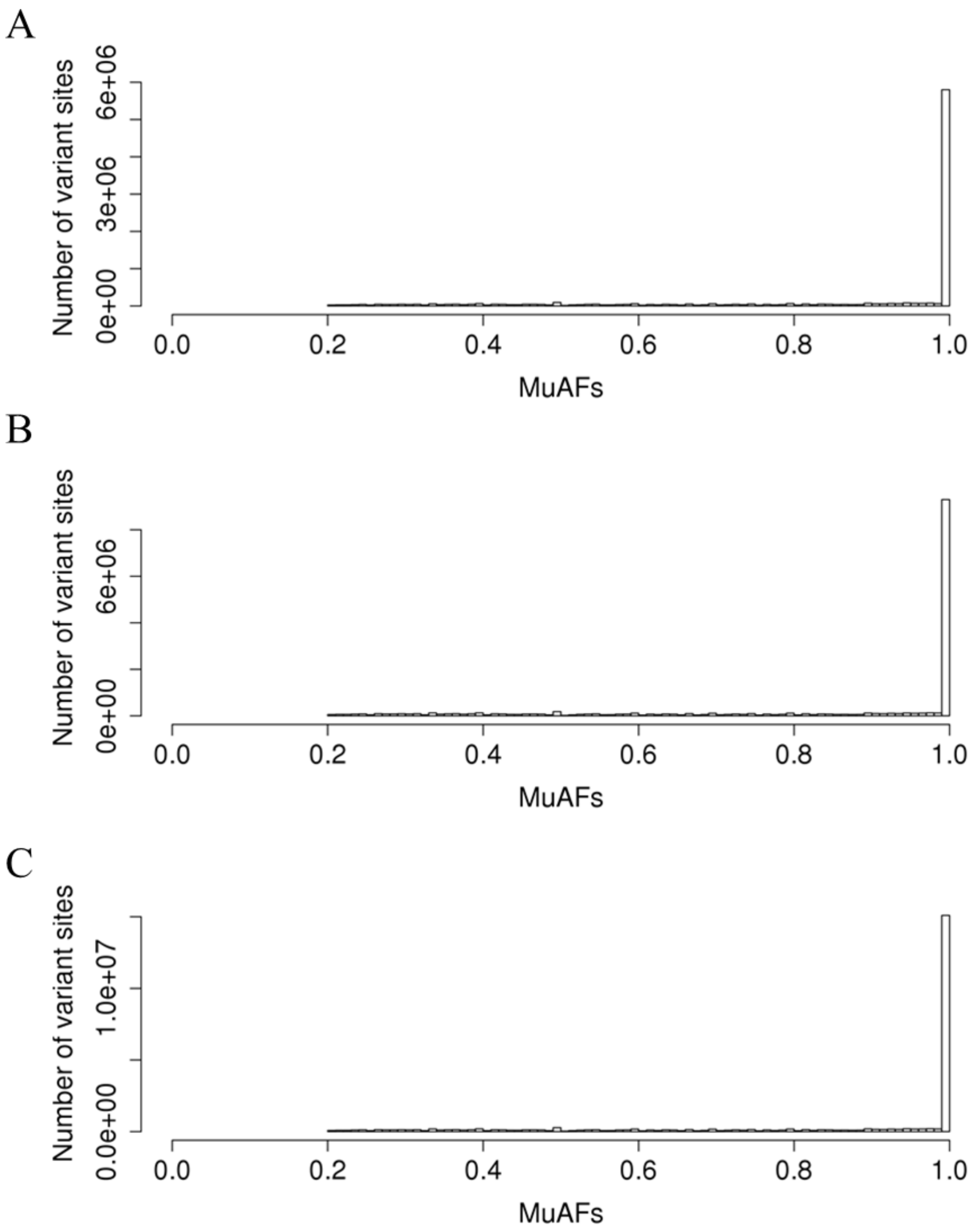


**Figure S4. Distributions of MuAFs at variant sites of *B. coprocola* in T2D (A), HC (B) and all (C) samples with sufficient NGS reads.**

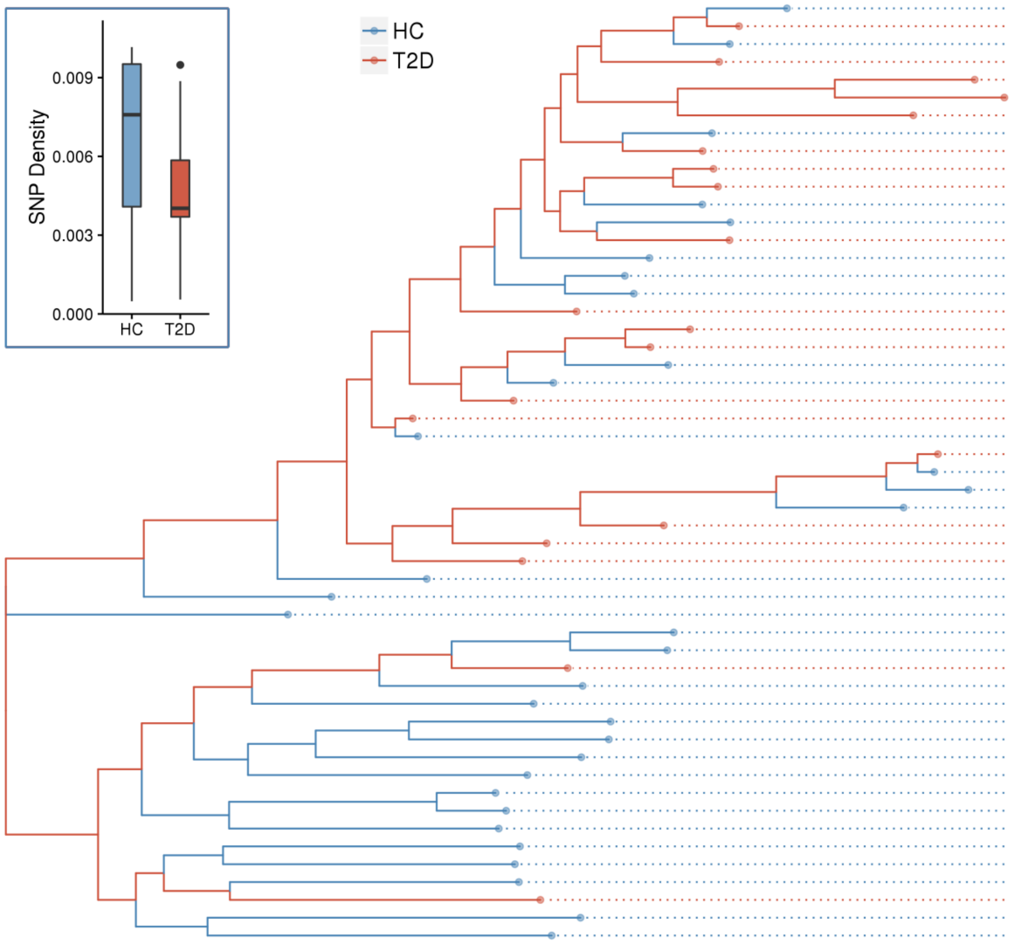


**Figure S5. Phylogenetic tree of *B. coprocola* strains based on variant sites with >0.8 MuAFs.**

The phylogenetic tree was built based on genome level SNPs with a >0.8 MuAF. The intra-tree-distance (quantified based on average pair-wise patristic distance) among T2D individuals' genomes (0.00775) was smaller than these of HCs (0.01085) and total samples (0.01032). Boxplot graph of variation density for *B. coprocola* in each sample group are shown in the inset graph.

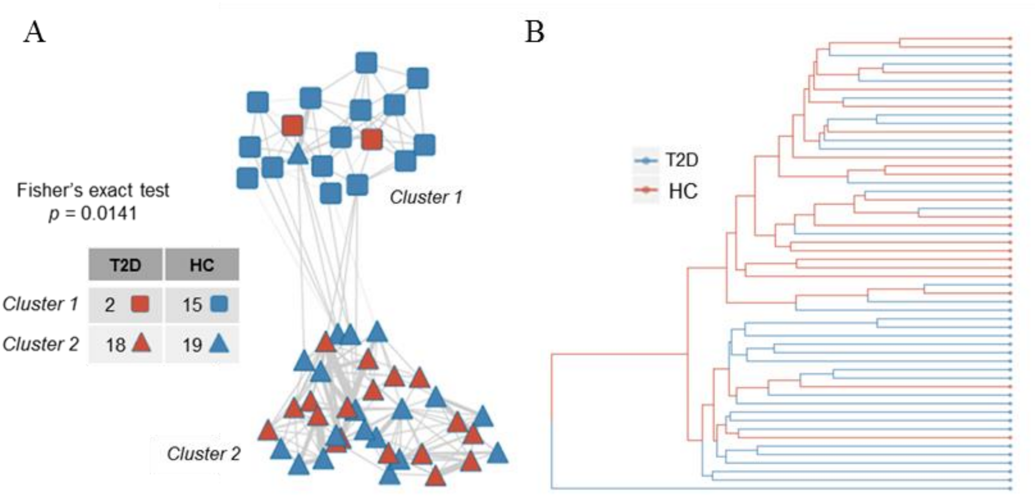


**Figure S6. Results of AP clustering and hierarchical clustering based on MuAFs of variant sites in *B. coprocola*.**

**A.** Samples were clustered into two clusters by AP clustering, and are illustrated a network. The width of edge denotes similarity between two samples. The 2×2 contingency table and *p* value of Fisher's exact test are shown up-left. **B.** Tree generated by hierarchical clustering. Red and blue branches indicate T2D samples and HC samples, respectively. The intra-tree-distances among T2D, HC and total samples are 150.17, 174.39 and 167.70, respectively.

A

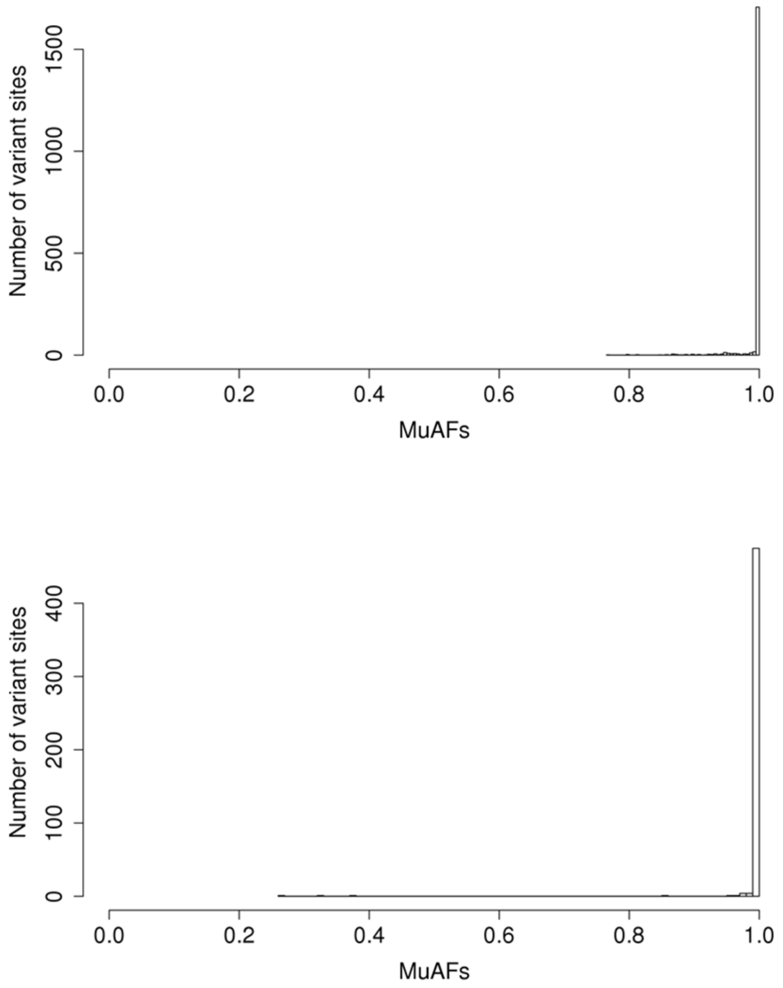


**Figure S7. Distributions of MuAFs at variant sites in *EDU99824.1* (A) and *EDUV02303.1* (B).**

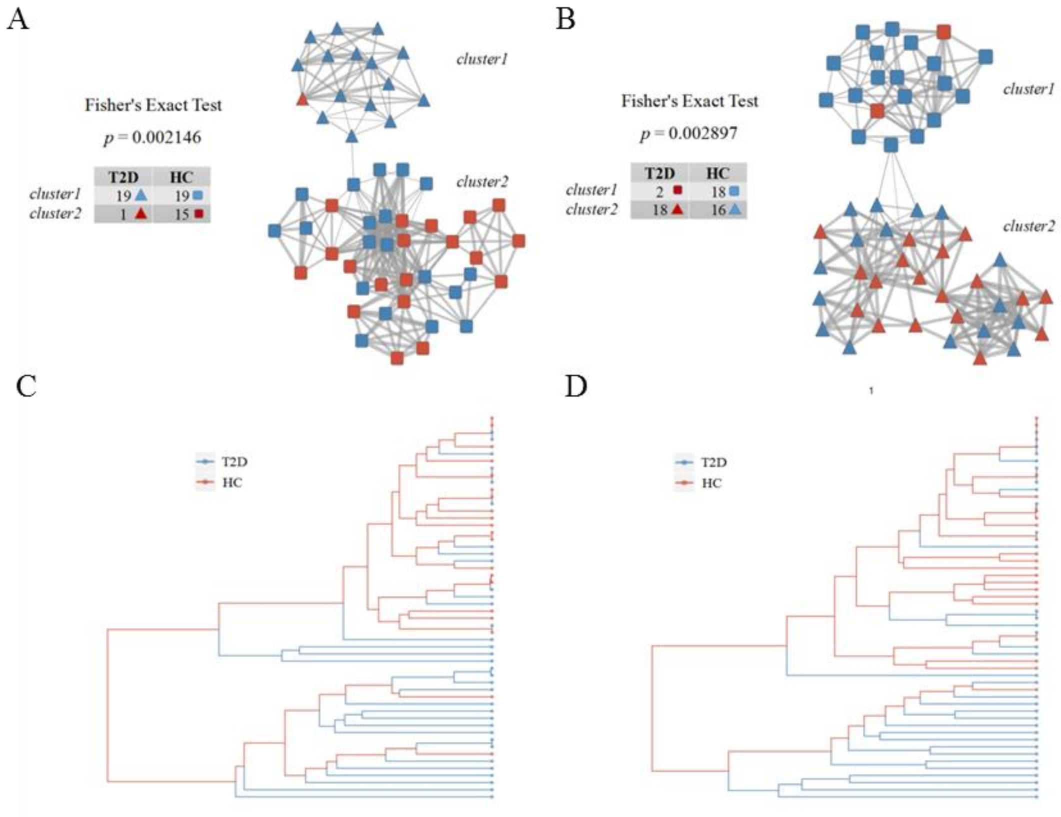


**Figure S8. Results of AP clustering and hierarchical clustering based on MuAFs of variant sites. A-B.** Samples were clustered into two clusters by AP clustering based on MuAFs of variant sites in *EDU99824.1* (**A**) and *EDV02303.1* (**B**). The width of edge denotes similarity between two samples. The 2×2 contingency table and *p* value of Fisher's exact test are shown up-left. **C-D.** Tree generated by hierarchical clustering based on MuAFs of variant sites in *EDU99824.1* (**C**) and *EDV02303.1* (**D**). Red and blue branches indicate T2D samples and HC samples, respectively. For *EDU99824.1*, the intra-tree-distances among T2D, HC and total samples are 4.14, 7.53 and 6.78, respectively. For *EDV02303.1*, the intra-tree-distances among T2D, HC and total samples are 4.84, 7.82 and 7.12, respectively.
